# The State Medical Association of Texas—Thirty-Sixth Annual Meeting

**Published:** 1904-03

**Authors:** 


### TEXAS MEDICAL JOURNAL.

AUSTIN, TEXAS.

A MONTHLY JOURNAL OF MEDICINE AND SURGERY.

EDITED AND PUBLISHED BY

F. E. DANIEL, M. D.

Mrs. F. E. DANIEL, Managing Editor.

ASSOCIATE EDITOR:

WITTEN BOOTH RUSS, M. D., San Antonio, Texas.

Published Monthly at Austin, Texas. Subscription price $1.00 a year in advance

Eastern Representative: John Guy Monihan, St. Paul Building, 220 Broadway, New York City.

Official organ of the West Texas Medical Association, the Houston District Medical Association, the Austin District Medical Society, the Brazos Valley Medical Association, the Galveston County Medical Society, and several others.

### THE STATE MEDICAL ASSOCIATION OF TEXAS—THIRTY-SIXTH ANNUAL MEETING.

### LOCAL ARRANGEMENTS.

**To Meet at Austin, April 26, 27, 28 and 29.**

Great enthusiasm has been awakened in the medical profession of the great State of Texas,—a regular shaking up of dry bones,—in consequence of the success or the reorganization of the State Medical Association. The total enrollment of active members now exceeds 2200, against 386 last year. It is confidently expected that there will be one thousand physicians in attendance at the great meeting to be held at the Capital of Texas the last week in April, and arrangements to entertain them, and the ladies who will accompany many of them, are being made. Oh, it will be a grand meeting, and will mark an epoch in the organization. A number of the distinguished physicians from other States, from the army and navy, and the United States Public Health Service, have been personally and especially invited, and it is hoped that some of the celebrities will be in attendance. Some notable papers will be presented, and it is expected that a large part of one day will be devoted to the discussion of yellow fever, and "quarantine or no quarantine;" to the subject of consumption; to that of railway sanitation, besides the subject of the papers read in the several sections.

An invitation is hereby cordially and earnestly extended to every reputable and progressive physician in Texas to come. A warm welcome will be given them, and they will be received in the ranks of progressive medicine with glad acclaim.

The social features of the meeting, too, will be of a high order, and full of delight. The local physicians and citizens feel a pride in the matter, and are going to entertain their guests in a distinguished manner. A grand reception, ball and supper—with oodles of champagne punch, and a stronger drink for those who want it—will be given at the Driskill, and when the music rises with its voluptuous swell, something will be doing "sho' nuff." Receptions will be given also at the residences of some of the local physicians, and, altogether, we can promise our guests—doctors and ladies—a delightful time.

The President and Faculty of the University of Texas have courteously placed the great auditorium of the University building at the services of the Association, together with room for the House of Delegates, the Judicial Council, and for the various committees, and also a hall for exhibits of books, pharmacals, surgical and other appliances, and the offer has been accepted. President Prather and the professors are on the reception and entertainment committees, and will co-operate with those having the program in charge to make everything go off with *eclat,* or like a sky rocket, with brilliancy, beauty and facility. There is much at the University to interest visitors: a splendid library, art halls, statuary and painting, the chemical, biological and physiological laboratories, etc., and time will be given for visits to these features.

As the University is some considerable distance from the business part of Austin and the hotels, arrangements will be made with the caterer who "feeds" the students (Brackenridge Hall, on the campus of the University) to furnish dinner there for as many as want it, at about 50 cents each, thus obviating the necessity of going to town to get dinner, and the delay of getting back. There are also several restaurants near the University. It has been arranged with the electric car management to carry the crowds to and from the University without hitch or delay.

Of course, there will be no charge for floor space for exhibits, and pharmacal, surgical and other manufacturers are urged to send their best men with a full lay out of their wares. It will be "worth while."

Special reductions in rates at hotels and boarding houses will be secured, and also a special round-trip rate on all railroads. These will be announced in due time by printed program, which will be a special feature of the April issue of the "Red Back." This number, bye the bye, will be an extra large edition, and a regular whizzer! Our representative will have a table in exhibit hall, with a display and plenty of blank receipts, and will be in a humor to take subscriptions with neatness and dispatch, at all hours. Every fellow is expected to have an extra dollar in his pocket for the contribution box of the "Red Back."

The Arrangement Committee—Dr. T. J. Bennett, Chairman, on behalf of the State Medical Association, and Dr. Ralph Steiner, President, on behalf of Travis County Medical Society—have appointed sub-committees as follows, and made the following "arrangements:"

*Entertainment.*—Dr. B. M. Worsham, Chairman.

*Reception.*—Dr. S. E. Hudson, Chairman.

*Transportation.*—Dr. H. B. Hill, Chairman.

*Hotels.*—Dr. J. S. Wooten, Chairman.

*Invitation*—Drs. Daniel, T. D. Wooten and Tabor.

*Finance*—Dr. M. M. Smith.

Invocation. Rev. Werlein, Pastor Tenth Street Methodist Church.

Adresses of Welcome. On behalf of the city, His Honor, Mayor White; Citizens, Rev. R. J. Briggs, M. D.; Medical Profession, Dr. F. E. Daniel; University, President Prather.

---

Conférence on Railway Sanitation.—A number of chief surgeons and superintendents of Texas railroads met in the office of State Health Officer Tabor in Austin, February 23d, to talk over Dr. Tabor's recent rules and regulations to govern railroad disinfection and general sanitation, in pursuance to the recently passed law on the subject. These rules and regulations were published in our January number. Amongst the surgeons were Dr. W. G. Jameson, Chief Surgeon of the I. & G. N. ("The Texas Railroad"), the only road, bye the bye, on which disinfection of cars is in operation; Dr. R. W. Knox, Chief Surgeon S. P. System; Dr. J. R. Stewart, of the H. & T. C.; Dr. C. A. Smith, of the Cotton Belt, and Dr. Scott, of the G., C. & S. F. Mr. Pettybone, Superintendent of the G., C. & S. F.; Mr. Van Vleck, Superintendent of the S. P. System, were here also. The date when the rules become effective is March 31st, as heretofore announced.

I can not see that anything was accomplished by the conference. I was not present, but, from the press reports, it seems that there was a general protest against having spittoons in the ladies' cars, as tending to encourage chewing in these cars. It was urged that cars entering Texas from other States, where there is no law to require it, are not provided with spittoons, and, as Texas lines take these cars at the State lines in some instances, they become liable for failure to provide them. Dr. Knox also thought "disinfection" of cars, after they enter Texas, would be unnecessary, if they were properly disinfected at point of departure, St. Louis, for instance. There is no disinfection of cars done at St. Louis, New Orleans or elsewhere, so far as I know. It is required by law only in Teaxs. Hence, that objection is not valid. Besides, the law regarding disinfection of coaches, and Dr. Tabor's rules to enforce it, are very vague and indefinite. They are to be disinfected "when they become infected." Now, who is to be the judge, and say when a day coach is infected? While with regard to sleepers, they are to be disinfected only "at the end of each run." It may be assumed that, as some of the roads protest against the enforcement of these rules, and object to doing more than the law requires, the disinfection, if practiced at all, will be of a perfunctory nature, and only sufficient to comply with the requirements and save themselves. It is to be assumed that cars in which consumptives travel are always infected, and Dr. Tabor should, and doubtless will, see that the disinfection is thorough and effective, or it will not be done. The railroads—some of them—are very much of Jay Gould's opinion, "the public be damned." They are out for revenue. Dr. Tabor will not prescribe the manner of disinfecting, nor the apparatus to be employed. It is generally understood, however, that formaldehyde must be used, as it is the only disinfectant suitable for sleepers, and at once effective and free from objection. Whatever plan or machine adopted by any road, must, however, have his approval.

To effectually fumigate a car in which tubercle bacilli are supposed to be lodged in the fabrics, formaldehyde gas should be used in vast volume, so as to penetrate. The use of sheets wet with formaline solution will not disinfect a car. It is, at best, only a deodorizer.

